# Adult-Type Rhabdomyoma of the Larynx in Birt–Hogg–Dubé Syndrome: Evidence for a Real Association

**DOI:** 10.1007/s12105-018-0922-6

**Published:** 2018-05-09

**Authors:** Ramkishan Balakumar, Matthew R. B. Farr, Malee Fernando, Ala Jebreel, Jaydip Ray, Sara Sionis

**Affiliations:** 1 Department of Ear, Nose and Throat, Royal Hallamshire Hospital, Sheffield Teaching Hospitals NHS Foundation Trust, Glossop Road, Sheffield, S10 2JF UK; 2 Department of Histopathology, Royal Hallamshire Hospital, Sheffield Teaching Hospitals NHS Foundation Trust, Sheffield, UK

**Keywords:** Birt–Hogg–Dube syndrome, Rhabdomyoma, *Folliculin*, Larynx

## Abstract

The autosomal dominant Birt–Hogg–Dubé syndrome is known to be associated with skin, lung and kidney lesions. It is caused by heterozygous germline mutations in the *folliculin* gene and has a high penetrance. We report the case of a 51 year old woman with Birt–Hogg–Dubé syndrome who presented with a laryngeal mass. Imaging confirmed a mass centered on the piriform sinus and following excision histological examination confirmed the lesion was composed of polygonal cells with abundant eosinophilic cytoplasm consistent with a rhabdomyoma. Laryngeal rhabdomyoma is rare condition and has not been previously described in association with Birt–Hogg–Dubé. In patients with Birt–Hogg–Dubé syndrome who develop upper aerodigestive tract symptoms secondary to mass lesion an adult-type rhabdomyoma might be considered as a differential, with endoscopic excision being the treatment of choice.

## Introduction

Birt–Hogg–Dubé (BHD) syndrome (MIM #135150) is a rare autosomal dominant condition predisposing sufferers to develop characteristic skin lesions (fibrofolliculomas, trichodiscoma and acrochordons), multiple lung cysts, spontaneous pneumothoraces and renal neoplasms [1]. In the head and neck region further associations with multinodular goitre, parotid-gland oncocytoma, tonsillar carcinoma [1] and parathyroid adenoma [2] have been reported. This syndrome is caused by heterozygous germline mutations in the *folliculin* gene (*FLCN*) located on chromosome 17p11.2 [1]. The functions of this gene are largely unknown but there is evidence to support a role as a tumour suppressor [1, 3].

Hamartomas such as rhabdomyomas are benign, focal malformations comprising of excess normal tissue growing in a disorganised fashion. A single ‘rhabdomyoma’ has been described among the cohort of 51 families with BHD syndrome, but its site was not indicated [4]. Since then, an adult rhabdomyoma in a presumed parathyroid adenoma and a cardiac rhabdomyoma in an infant carrying a *FLCN* mutation have been described [5]. We report an adult laryngeal rhabdomyoma in a patient with BHD syndrome.

## Case Report

A 51 year old female with BHD syndrome was noted to have a soft fleshy mass in her supraglottic region during intubation for an open partial nephrectomy for a renal tumour. This operation was uneventful and pathology confirmed 11 chromophobe renal carcinomas (sizes ranging from 2 to 10 mm) and a 9 mm renal cell carcinoma with hybrid chromophobe—oncocytoma histology.

She was asymptomatic with regards to the mass in her larynx and had no cervical lymphadenopathy. BHD had been confirmed 3 years prior to her surgery by genetic testing which demonstrated a 4 bp deletion in one copy of her FLCN gene. She had no other significant past medical history. She had no first degree relatives with confirmed BHD however had a brother who had been recently diagnosed with a renal tumour and was awaiting a partial nephrectomy. Flexible nasendoscopy in ENT outpatient clinic demonstrated a smooth mass around the right aryepiglottic fold and right arytenoid, obscuring the right piriform fossa. It was noted she had reduced movement of the right vocal cord.

**Fig. 1:**
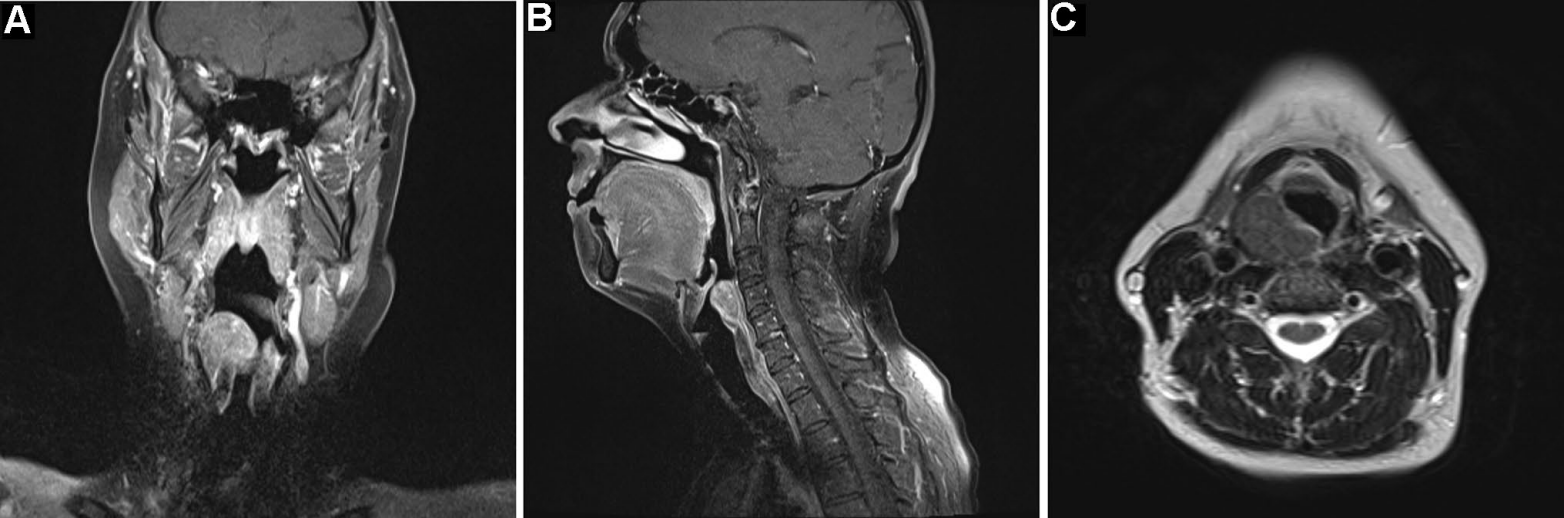
MRI scans with contrast demonstrating an enhancing mass centered on the right piriform fossa **a** T1-weighted coronal, **b** T1-weighted sagittal, **c** T2-weighted axial

An MRI neck with contrast delineated a 28 mm diameter mass centred on the right piriform fossa and extending into the right supraglottis without associated lymphadenopathy (Fig. 1). Clinical and radiological features were suggestive of a benign nature and, consequently, transoral laser microsurgery was planned.

**Fig. 2:**
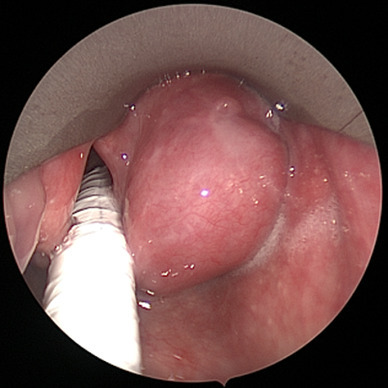
Endoscopic photograph of laryngeal mass in the right piriform fossa taken at the time of surgery

The patient underwent a microlaryngoscopy under general anaesthetic. The mass appeared to be pedicled on the right aryepiglottic fold and the medial wall of the right piriform fossa. A 3 cm submucosal lobulated soft mass was excised using Co_2_ laser (Sharplan) set on 4 W, continuous wave in super-pulse mode under microscopic vision with a focal length of 400 mm. (Fig. 2). The postoperative period was uneventful and the patient was discharged on the day of the operation with improved motility of the right vocal cord.

**Fig. 3:**
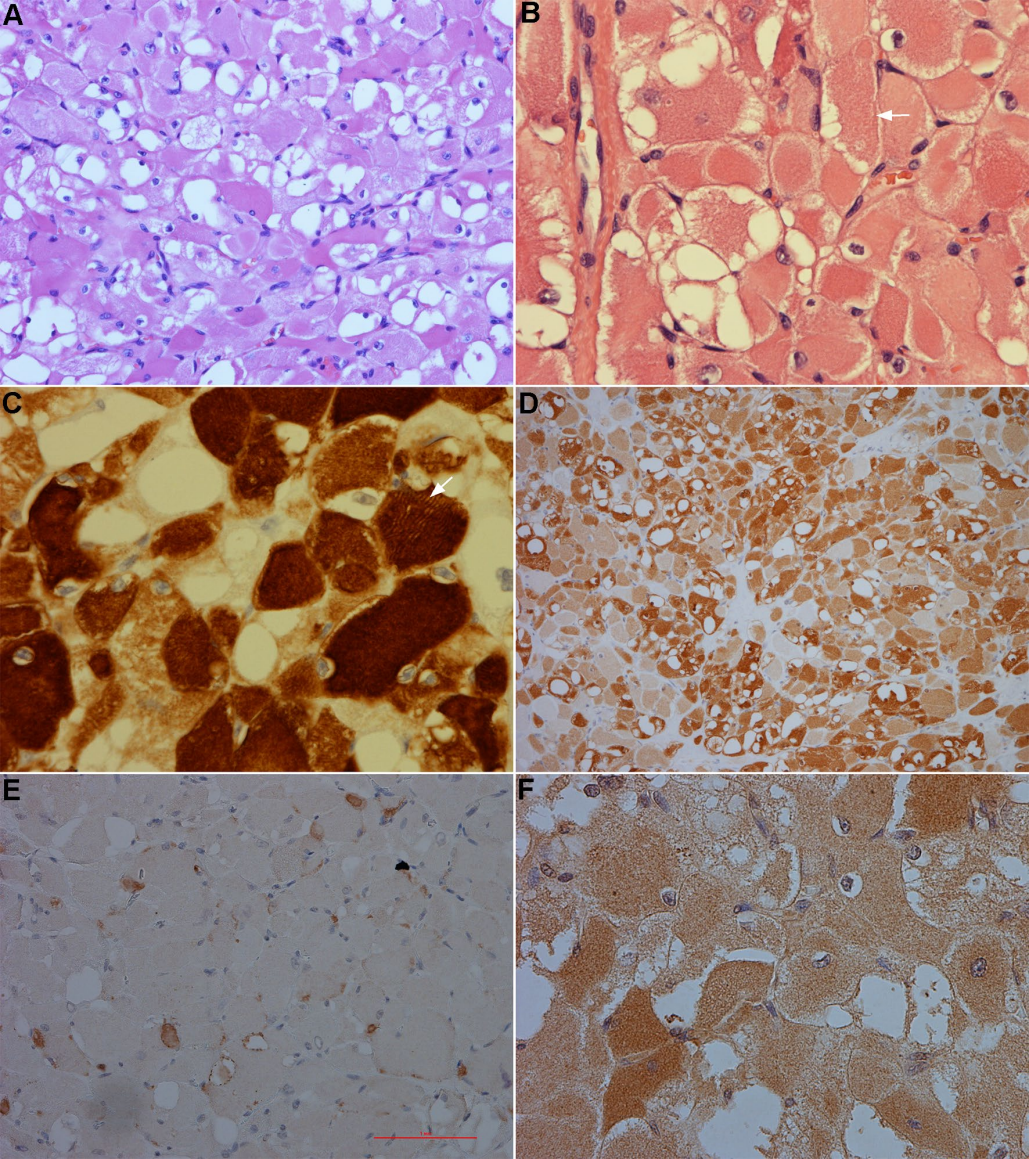
**a** Rhabdomyoma composed of polygonal cells with abundant eosinophilic cytoplasm, bland nuclei and scanty nucleation. Haematoxylin and Eosin ×200. **b** Rhabdomyoma with cells demonstrating cross striations (arrow). Haematoxylin and Eosin ×400. **c** Rhabdomyoma, with immunohistochemistry confirming expression of desmin with evidence of cross striations (arrow) ×400. **d** Rhabdomyoma, with a few scattered nuclei expressing S100 × 100. **e** Rhabdomyoma showing patchy expression of myogenin ×200. **f** Rhabdomyoma stained for MyoD1 expression ×400

Histological examination confirmed the lesion was completely excised with good margins. It was composed of polygonal cells with abundant eosinophilic cytoplasm (Fig. 3a, b). Some of the cells demonstrated cross striations (Fig. 3b, c arrow), but no obvious crystalline cytoplasmic inclusions were seen. There were no myxoid areas, mature skeletal muscle fibres, atypia, necrosis or mitosis. Desmin expression was extensive and highlighted the cross striations, a feature not seen in the main differential diagnosis of a granular cell tumour (Fig. 3c). S100 highlighted a few scattered nuclei expressing this marker, as seen in adult rhabdomyoma (Fig. 3d). Myogenin showed patchy expression (Fig. 3e). Follow up has confirmed no evidence of recurrence on flexible nasendoscopy 18 months after initial resection.

## Discussion

A rhabdomyoma is a form of hamartoma that may occur at cardiac and extracardiac sites. These may be further sub-classified histologically into adult and fetal types depending on the level of differentiation [6, 7]. Adult rhabdomyoma show mature skeletal muscle differentiation. The majority of extra-cardiac rhabdomyomas occur in the head and neck region (around 70%), mainly affecting the upper aero-digestive mucosa (pharynx, oral cavity and larynx) and soft tissue of the neck [6–9]. It is thought that these originate from the third and fourth branchial musculature due to its close association with the neck [9]. Deregulation of the sonic hedgehog pathway has been suggested to be important in the pathogenesis of rhabdomyoblastic tumours [10]. The clinical presentation depends on the location with dysphagia, hoarseness or respiratory distress typical of aerodigestive tract involvement. Around 10% are asymptomatic; the median age of presentation is 60 years (ranging from 33 to 80 years) with a male predilection (3:1 ratio) [6, 7]. Our patient was a 51 years old female and given the rarity of extra-cardiac rhabdomyomas, a comparison of prevalence to those without this syndrome would be of little clinical value.

Macroscopically adult rhabdomyomas are a deep tan to red-brown mass growing as a solitary (70%) or multinodular lesion (26%) with discrete nodules. Rarely, they may be multicentric (4%) [6, 7]. The histological differential diagnoses include granular cell tumours, hibernomas, oncocytomas and paragangliomas. Immunohistochemical staining is helpful in distinguishing between these diagnoses. Adult rhabdomyoma staining will characteristically confirm skeletal muscle differentiation, with tumours positive for myoglobin, desmin and muscle-specific actin [11, 12]. S-100 expression, when present, is weak and focal [13]. Complete surgical excision is recommended because local recurrence is related to incomplete clearance [6, 7, 9].

A ‘rhabdomyoma’ has been described among the cohort of 51 families with BHD syndrome assessed by Toro et al. [4], but its site was not indicated. Since then, there have been few reports of AR with BHD syndrome. One case of AR in a parathyroid adenoma has been described in a patient undergoing thyroidectomy and parathyroidectomy for toxic multinodular goitre and primary hyperparathyroidism [14]. Cardiac rhabdomyomas have been described in the Nihon rat model of BHD [15]. Though rhabdomyomas have been described in association with BHD, there has not been a report in the English literature of a laryngeal lesion associated with the condition.

Tuberous sclerosis (TSC) is a tumour suppressor gene syndrome and its clinical similarities to that of BHD suggest that the FLCN and TSC proteins may function in a common cellular pathway [16]. Cardiac rhabdomyomas in patients with TSC are linked to aberrant mTOR signalling [17] through the loss of regulation by the hamartin (TSC1) and tuberin (TSC2) complex. Though the function of tumour-suppressor protein folliculin (FLCN) is still being confirmed, the identification of the FLCN-interacting protein, FNIP1 has generated much interest and could explain the association with rhabdomyomas. FNIP1 interacts with 5′AMP-activated protein kinase (AMPK), a key molecule for energy sensing that negatively regulates mTOR activity, suggesting that FLCN may be regulated by mTOR and AMPK signaling [1, 17]. This has lead Bondavalli et al. to raise the possibility that mutations in *FLCN* might be the cause of cardiac rhabdomyomas and argue for BHD to be included in the differential diagnosis of these hamartomas [5].

Expression of *FLCN* mRNA has been demonstrated widely, but not universally, in a variety of tissues with expression limited to certain cell types. This selective expression suggests the gene may be involved in particular cell processes [1, 3]. Warren et al. performed a study which looked at the level of *FLCN* expression in these tissues. Although the larynx was not mentioned in this study other tissues including tonsils, lymph nodes, bladder, myometrium, pancreas and parotid gland showed the mRNA expression [3]. The finding of an adult rhabdomyoma within the piriform fossa also raises questions as to the role of folliculin in the hypopharynx.

## Conclusion

It is possible that the presence of *FLCN* mutations in patients with hamartomas is more common than is widely known but the limited awareness of BHD is resulting in cases going unrecognised. The few reports describing an association between AR and BHD, whilst insufficient evidence for a causal link between the two conditions, point towards a predisposition to hamartoma formation associated with haploinsufficiency of *FLCN*. Amongst patients with BHD, an adult-type rhabdomyoma might be considered in the differential diagnosis when a mass lesion arises in an unexpected site such as the upper aerodigestive tract. Should treatment of an upper aerodigestive tract rhabdomyoma be required endoscopic excision should be the intervention of choice.
